# Une scoliose révélant un ostéome ostéoïde

**DOI:** 10.11604/pamj.2013.14.58.1538

**Published:** 2013-02-11

**Authors:** Abdelhalim Mahmoudi, Khalid Khattala, Mohamed Rami, Aziz Elmadi, Chater Lamiae, Bouabdallah Youssef, My Abderrahmane Afifi

**Affiliations:** 1 Service de Chirurgie Pédiatrique CHU Hassan II de Fès, Maroc

**Keywords:** Scoliose, douleur, rachis, ostéome osteoide, Scoliosis, pain, spine, osteoid osteoma

## Abstract

L'ostéome ostéoïde rachidien est une lésion rare. Nous rapportons un cas d'ostéome ostéoïde rachidien chez une fille, qui consultait pour une scoliose raide douleureuse partiellement calmée par la prise de salicylés. La tomodensitométrie (TDM) centrée sur la région dorsolombaire montrait une lésion typique d'ostéome ostéoïde. Un curetage biopsique de la tumeur a été realisé par voie postérieure. L'anatomo-pathologie confirmait le diagnostic d'ostéome ostéoïde. L’évolution était favorable au recul de 3 ans.

## Introduction

L'ostéome ostéoïde (OO) a été décrit pour la première fois par Jaff [1] en 1935. Il s'agit d'une tumeur ostéoblastique bénigne qui comporte une petite lésion centrale charnue très vascularisée ostéoïde et immature (le nidus) entourée d'une ostéocondensation réactionnelle. Elle est découverte le plus souvent chez l'adolescent et l'adulte jeune. Cette lésion est située préférentiellement au fémur proximal. Les localisations rachidienne reste rare, elle représente seulement 10% des cas. Nous rapportons le cas d'un ostéome ostéoïde rachidien révélé par une scoliose raide et douloureuse chez une adolescente de 13 ans.

## Patient et observation

On a eu le consentement du tuteur légal du patient. De ce fait la publication de cette observation ne pose aucun problème pour notre patient ou bien pour ses proches.

SM fille agéé de 13 ans, sans antécédents particuliers, est vu en consultation pour scoliose douleureuse. Depuis 3 ans, elle se plaint de lombalgies, de type inflammatoire mise sous antinflammatoires non steroidiens avec amelioration transitoire. L'examen trouve une scoliose lombaire gauche avec contracture des masses paravertébrales, une gibosite avec une hauteur de 1cm, des douleurs de la région lombaire à la palpation. IL n'y a ni syndrome moteur déficitaire ni syndrome pyramidal irritatif, pas de trouble vésicosphinctérien, pas de signe méningé. Le reste de l'examen somatique est normal. La biologie (NF, VS, CRP) n'est pas en faveur d'un processus infectieux ou inflammatoire.

**Figure 1:**
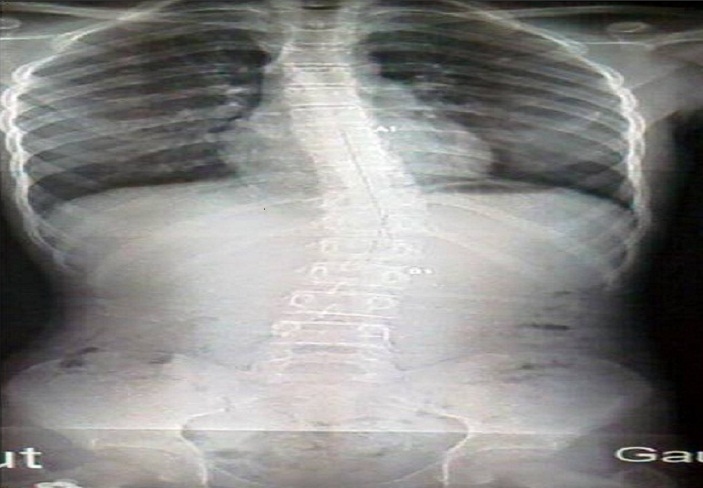
La radiographie standard du rachis de face: objective une scoliose D8-L2 gauche de 25

**Figure 2:**
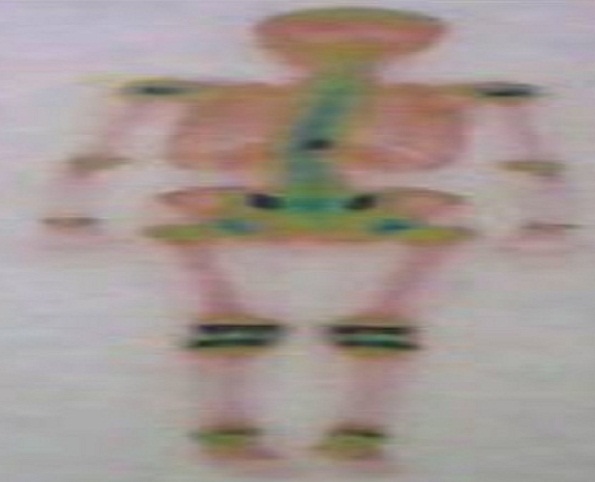
La scintigraphie osseuse (montre une volumineuse hyperfixation de la lame vertébrale droite de D11

**Figure 3:**
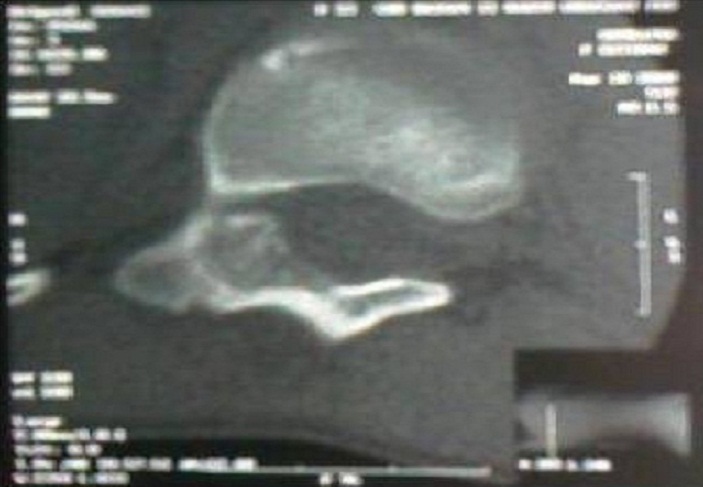
L'examen tomodensitometrique du rachis met en évidence une image lacunaire à contours denses, de la lame vertébrale droite de D11 faisant évoquer un ostéome ostéoïde

La radiographie standard (Figure 1) avait objectivé une scoliose D8-L2 gauche de 25. La scintigraphie osseuse (Figure 2) avait montré montre une volumineuse hyperfixation de la lame vertébrale droite de D11. L'examen tomodensitometrique (Figure 3) du rachis met en evidence une image lacunaire à contours denses, de la lame vertébrale droite de D11 faisant évoquer un ostéome ostéoïde.

La patiente a été opérée par voie postérieure avec curetage de la tumeur emportant l'articulaire et la lame latérale de D11. Le défect a été comblé par de l'os spongieux prélevé au niveau de la crête iliaque avec immobilisation par corset pendant 3 mois. Les suites postopératoires étaient simples, avec disparition immédiate de la douleur. A 3 ans de recul, la patiente présente un rachis indolore et une récupération totale de la mobilité, avec disparition de la scoliose.

## Discussion

L'ostéome ostéoïde (O.O), tumeur osseuse bénigne décrite pour la première fois en 1935 par Jaffé [1], est la cause la plus fréquente de douleurs osseuses nocturnes calmées par les salycilates au cours des trois premières décades de vie. L'ostéome ostéoïde survient essentiellement chez les sujets jeunes de sexe masculin avec un sexe ratio de 3/1[2]. Cette tumeur représente 12% de toutes les tumeurs bénignes de l'os [2]. Elle est de siège ubiquitaire néanmoins sa localisation au niveau des os des membres inférieurs est la plus commune. La localisation au niveau du rachis représente 10% des cas [2].

Les manifestations cliniques sont le plus souvent typiques, à type de douleurs nocturnes, insomniantes, totalement disproportionnées par rapport à la taille de l'O.O et calmées par la prise de salicylés [3–5]. L'image radiologique classique ne se retrouve pas davantage dans notre cas. Comme on a pu le voir, il a été nécessaire de recourir à la tomodensitométrie et la scintigraphie pour obtenir un diagnostic. En effet, la radiographie standard ne donne le diagnostic d'ostéome ostéoide que dans 75% des cas. Les radiographies standards réalisées chez notre patient étaient normales du fait du siège et de la petite taille de la lésion (< 1cm). La scintigraphie est plus sensible que la radiologie conventionnelle. Sa négativité élimine pratiquement le diagnostic d'ostéome ostéoïde. Si elle est positive, elle montre une hyperfixation marquée par le nidus [6]. En fait, la TDM permet l'identification et la localisation de la tumeur avec une grande sensibilité [7]. Elle montre une lésion hypodense, bien limitée, contenant ou non une calcification centrale, cernée par un liseré d'ostéocondensation et mesurant moins de 10 mm de diamètre [6]. L'IRM est très sensible mais non spécifique dans le diagnostic des ostéomes ostéoïdes rachidiens du fait de la petite taille de la tumeur, de son siège au niveau des arcs postérieurs des vertèbres, et du signal hypo-intense du nidus calcifié sur toutes les séquences [8, 9]. L'ostéome ostéoïde en IRM se présente sous différents aspects selon la vascularisation du nidus. Il est généralement en hyposignal T1, hypersignal T2 avec une ostéosclérose réactionnelle périphérique. L'IRM est surtout sensible dans la détection de l'atteinte des parties molles adjacentes à la lésion. La tomodensitométrie est plus performante dans la détection des calcifications et de l'atteinte des corticales osseuses.

Le diagnostic différentiel majeur au niveau du rachis est l'ostéoblastome. L'histoire clinique, le siège, l'aspect radiologique et surtout la taille permettent, dans la majorité des cas de faire la part entre ces deux entités. L'ostéoblastome a une taille qui dépasse 2cm. Chez notre patient, l'aspect radiologique, la petite taille de la lésion et l'aspect histologique concordent avec le diagnostic d'ostéome ostéoïde.

Le traitement médical, la chirurgie d'exérèse, l'ablation percutanée, ou la radiofréquence sont les procédés thérapeutiques les plus utilisés [10]. Le traitement de choix de l'ostéome ostéoïde spinal est la résection tumorale complète par une voie postérieure [11, 12]. On a opté pour la résection chirurgicale de la tumeur à cause de son siège superficiel, et car le traitement percutané, soit par forage résection sous contrôle TDM, soit par thermocoagulation, est difficile, De plus, le traitement percutané ne permet pas la confirmation histologique du diagnostic [13, 14].

L’évolution se fait vers la guérison si l'ablation du nidus est complète [15].

## Conclusion

La découverte d'une Scoliose douloureuse chez un enfant doit conduire à un bilan étiologique comprenant en première intention une scintigraphie osseuse. L'existence d'une hyperfixation localisée à l'isthme doit conduire pour nous à la réalisation d'une TDM dont l'intérêt est à la fois diagnostique et thérapeutique. L'ostéome ostéoïde doit être évoqué devant des scolioses douloureuses avec une hyperfixation isthmique localisée.
